# Diagnosis of prolonged grief disorder among older adults: mapping physicians’ knowledge and practice, and evaluating the effectiveness of a brief targeted training program

**DOI:** 10.3389/fpsyt.2026.1835855

**Published:** 2026-06-19

**Authors:** Alexander Manevich, Daphna Shefet, Yael Orion, Nili Elior

**Affiliations:** 1 Department of Clinical Psychology of Adulthood and Aging, Ruppin Academic Center, Kfar Monash, Hefer Valley, Israel; 2 School of Psychological Sciences, Faculty of Social Sciences, International Laboratory for the Study of Loss, Bereavement and Human Resilience, University of Haifa, Haifa, Israel; 3 Geriatric Psychiatry Services, Shalvata Mental Health Center, Hod HaSharon, Israel; 4 Department of Psychiatry, Gray Faculty of Medical and Health Sciences, Tel Aviv University, Tel Aviv, Israel; 5 Sharan – Medical Care at Home, Bnei Brak, Israel; 6 Clalit Health Services, Tel Aviv, Israel; 7 Department of Family Medicine, Gray Faculty of Medical and Health Sciences, Tel Aviv University, Tel Aviv, Israel

**Keywords:** prolonged grief disorder (PGD), elderly mental health, physician knowledge and training, diagnostic assessment, clinical implementation, ICD-11, DSM-5-TR

## Abstract

**Background:**

Prolonged Grief Disorder (PGD) is a psychiatric diagnosis with significant implications for individuals and society. Familiarity with its diagnostic criteria and recommended interventions is essential for early detection and effective treatment. As aging naturally involves multiple losses, older adults are at distinct risk of developing PGD, a condition associated with psychological and physical comorbidities and increased mortality. In the Israeli context, this issue holds particular significance following the events of October 7, 2023, and the ensuing war, which have had — and continue to have — a profound impact on the population’s mental health. Given PGD’s recent inclusion in diagnostic manuals, many physicians may lack sufficient knowledge, leading to under-recognition or misdiagnosis. This gap is particularly evident among older adults, for whom the assessment and management of psychological distress are often underassessed and undertreated, highlighting the urgent need for targeted training and broader integration of existing knowledge into clinical practice. Accordingly, the present study has two main objectives: (1) to assess physicians’ knowledge and use of PGD diagnostic criteria with older patients, and (2) to evaluate the effectiveness of a brief informational intervention on their understanding and diagnostic application.

**Methods:**

Using a mixed experimental design, the study will include senior physicians and residents specializing in geriatrics, psychiatry, and family medicine. Data will be collected via an online structured questionnaire containing professional background items and clinical vignettes designed to assess participants’ knowledge and application of PGD diagnostic criteria. Afterwards, participants will be individually randomized (1:1) via a computer-generated sequence in a partially participant-blinded, assessor-blinded controlled trial to either an experimental group viewing an informational PGD video or a control group viewing a structurally equivalent video (matched in length, graphics, and word count) addressing bereavement in older adults without directly referencing PGD. All participants will then complete the vignettes again to evaluate the effects of the intervention. A one-month follow-up assessment will evaluate the retention of intervention effects.

**Discussion:**

This first systematic Israeli study on PGD implementation will provide an empirical basis for policy development, professional training, and prevention programs. Its findings may enhance diagnostic accuracy, improve care efficiency, reduce systemic costs, and support ongoing evaluation and refinement of clinical practices.

**Clinical trial registration:**

## Introduction

Prolonged Grief Disorder (PGD) is a mental health condition recently included in the ICD-11 (1) and DSM-5-TR (2). It is characterized by unique core symptoms, including intense yearning for the deceased, persistent preoccupation with the deceased, or both. These symptoms are commonly accompanied by emotional pain, disruption of personal identity, loss of meaning and purpose in life, and impaired daily functioning (3). PGD is distinct from other psychiatric diagnoses such as Major Depressive Disorder, Generalized Anxiety Disorder, and Post-Traumatic Stress Disorder, and is associated with a range of negative outcomes, including physical health problems (4), increased risk of suicidal ideation or attempts, reduced life satisfaction, functional impairment, and greater utilization of health services. Epidemiological studies indicate that among bereaved populations, the prevalence of PGD is relatively low in representative samples (approximately 3–4%), while higher rates, up to 16%, are reported in non-probability or convenience samples (5).

Aging is naturally accompanied by experiences of loss and bereavement, and as individuals they encounter an increasing number of losses, including traumatic ones and various life-course separations. Consequently, older adults are considered at elevated risk for disruptions in the normative grief process in general, and for the development of PGD in particular (6, 7). Supporting evidence includes, for example, a meta-analysis of over 60,000 participants, which identified spousal loss and pre-death grief (experienced while caring for a terminally ill relative) as two major risk factors for PGD (8). Both phenomena, whose prevalence increases with age, illustrate older adults’ heightened vulnerability to the loss of significant others and its adverse consequences. Additionally, multiple studies have linked the loss of a close person, especially a spouse among older adults, to serious health outcomes, including immune dysfunction, increased cardiovascular risk, chronic pain, and even elevated mortality rates [e.g (9–11).

These findings indicate that PGD not only impacts individual quality of life but also imposes a significant economic burden on healthcare systems. Additional costs include medical treatments, medications, hospitalizations, and mental health services, alongside impaired daily functioning and productivity. Early identification and tailored intervention can therefore improve patient outcomes while reducing systemic costs [e.g (12–14).

Consistent with the gap between academic research and its systematic application in clinical practice (15), healthcare providers’ knowledge of complications in the normative grief process is often limited (16). Furthermore, given that PGD is a relatively new diagnosis not yet widely recognized among clinicians, raising awareness among physicians and mental health professionals is crucial for enabling early identification and appropriate intervention (6). This issue is especially relevant for older adults, given the evidence of underdiagnosis and undertreatment of psychological distress in this population [e.g (17, 18).

For example, although suicide rates among older adults in Israel and globally are higher than in any other age group, epidemiological studies show that the prevalence of clinically significant depression and anxiety is comparatively lower in older adults than in younger populations. Moreover, older adults with depression often report fewer typical cognitive-emotional symptoms (e.g., dysphoria, guilt, or feelings of worthlessness) and instead emphasize physical symptoms such as fatigue, somatic complaints, and difficulties with memory and concentration (19).

In fact, research indicates that many older adults who engaged in suicidal behavior had visited a physician shortly beforehand, yet their psychological distress remained undetected. This phenomenon stems, in part, from a lack of knowledge and appropriate skills among healthcare professionals regarding assessment and treatment in older populations. Additionally, research indicates the presence of ageist attitudes among clinicians, manifesting, for example, in reduced confidence in the effectiveness of interventions for the elderly. At the same time, many older adults avoid seeking mental health care for generational, social, personal, or practical reasons, making the primary care physician often the sole gateway to identifying psychological distress and referring patients for appropriate care (19). These findings highlight the need for targeted professional training to enhance understanding of the clinical characteristics unique to older adults and to facilitate effective, tailored intervention.

In the context of PGD, as noted earlier, early identification and diagnosis are crucial for prevention, improving quality of life, and reducing systemic burden and costs, as timely detection of the disorder can prevent psychological and physical deterioration and minimize unnecessary interventions. Additionally, misdiagnosis can also lead to inappropriate pharmacological treatment rather than evidence-based psychological intervention — particularly grief-focused cognitive-behavioral therapy (20), which is considered the treatment of choice for PGD (5). For example, a randomized controlled trial provided empirical evidence that PGD-focused psychotherapy effectively reduces grief symptoms, whereas citalopram (a selective serotonin reuptake inhibitor [SSRI]) did not have a significant impact on grief symptoms but did alleviate depressive symptoms in patients with comorbid depression (21). Moreover, due to PGD’s addiction-like features, recent research has focused on reward system activity in the brain, which plays a central role in the disorder (22).

In summary, PGD is a psychiatric disorder with significant implications for individuals and society. Comprehensive familiarity with its diagnostic criteria and recommended interventions is essential for early identification and optimal treatment. Aging, accompanied naturally by multiple losses, places older adults at a distinct risk for PGD, which is associated with increased mental and physical comorbidity and mortality. In Israel, this issue has gained particular significance following the events of October 7, 2023, and the subsequent war, which continue to critically affect the population’s mental health [e.g (23, 24). Since PGD was only recently included in diagnostic manuals, it is likely that many physicians are not yet sufficiently familiar with it. As a result, cases of under recognition, partial, or even incorrect diagnosis of the disorder may occur in clinical practice, leading to interventions that do not adequately address its unique characteristics. This gap is particularly pronounced among older adults, for whom the assessment and management of psychological distress are often suboptimal, underscoring the need for targeted training and broader integration of existing knowledge into clinical practice.

### Aims

The current study has two primary objectives: (1) to assess physicians’ knowledge and application of PGD diagnostic criteria in older adults — specifically geriatricians, psychiatrists, and family physicians; (2) to examine the short- and long-term impact of a brief, focused PGD intervention on physicians’ knowledge and diagnostic application.

### Expected results

The *primary outcomes* of the study are twofold. First, participants are expected to report significant knowledge gaps regarding PGD diagnostic criteria, reflecting difficulty or uncertainty in applying these criteria with older bereaved patients. Second, exposure to concise PGD-related information via a video-based intervention (with a control group viewing an equivalent video; see Methods) is expected to improve knowledge and application of diagnostic criteria. Specifically, correct responses on the clinical vignettes are expected to increase in both groups following the intervention, with a greater improvement in the experimental group compared to the control group. These improvements are expected to persist at one-month follow-up, particularly in the experimental group.

The *secondary outcome* concerns differences between medical specialties. Psychiatrists are expected to demonstrate higher baseline knowledge and application of PGD criteria compared to other participants, achieving a significantly higher proportion of correct responses on the clinical vignettes.

## Methods and analysis

### Participants

The study will employ a mixed experimental design. The target population includes senior physicians and residents in geriatrics, psychiatry, and family medicine working in diverse settings, including hospitals, clinics, geriatric centers, and private practices, who have frequent contact with older adults. Participation will be anonymous, voluntary, and based on informed consent. The sample will be recruited via convenience sampling through professional channels (e.g., mailing lists of relevant medical associations, professional social networks, and distribution via department and center administrators), aiming to ensure balanced representation across medical specialties and levels of professional training.

*Sample size:* Power analysis using G*Power (version 3.1) indicated that to detect a medium effect size (partial η² = 0.06) with 80% power and α = 0.05 in a mixed repeated-measures design with two independent variables, a minimum sample of 34 participants is required. To detect a small effect size (partial η² = 0.01) under the same conditions, 198 participants are needed. Accordingly, the study aims to recruit a sample size within this range.

### Measures

Data will be collected via an online structured questionnaire developed specifically for this study. The questionnaire has two main sections. The first includes nine closed-ended items (multiple-choice questions) assessing participants’ general background and experience working with bereaved patients. The second presents seven brief, hypothetical clinical vignettes created for this study to measure knowledge and application of PGD diagnostic criteria. For each case vignette, participants will be asked a closed-ended question, indicating whether they believe the case is likely to meet the diagnostic criteria for PGD or not (Study Protocol Appendices).

*Intervention:* After completing the initial questionnaire, half of the participants (experimental group) will view a 3-minute informational video providing concise evidence-based material on PGD, including core diagnostic criteria and clinical applications. The other half (control group) will view a 3-minute video presenting general information on the importance of promoting research, diagnosis, and tailored care for older adults experiencing bereavement, without reference to PGD. Videos are structurally matched in terms of graphics, duration, and word count to control for potential confounding variables. The rationale for presenting the explanatory material through a video rather than written text is grounded in empirical findings indicating that video-based instruction enhances learning effectiveness, particularly in the acquisition of clinical skills among healthcare professionals [e.g (25, 26) This advantage is attributed, among other factors, to the increased engagement, concentration, and interest elicited by video presentations compared to traditional text-based materials.

### Procedure

Ethical approval for the study was obtained from the Ruppin Academic Center Institutional Ethics Committee and the Helsinki Committee of Clalit Health Services. Participants will receive online invitations, including an explanation of the study purpose, participation procedure, and assurance of full anonymity. It will also be emphasized to participants that the questionnaire contains hypothetical scenarios intended solely to assess professional responses and does not include personal questions or require emotional disclosure. However, it will be clarified that the content may evoke uncomfortable feelings, and that participation can be discontinued at any point without any consequences. Participation will be voluntary and contingent upon providing informed consent.

**Figure 1:**
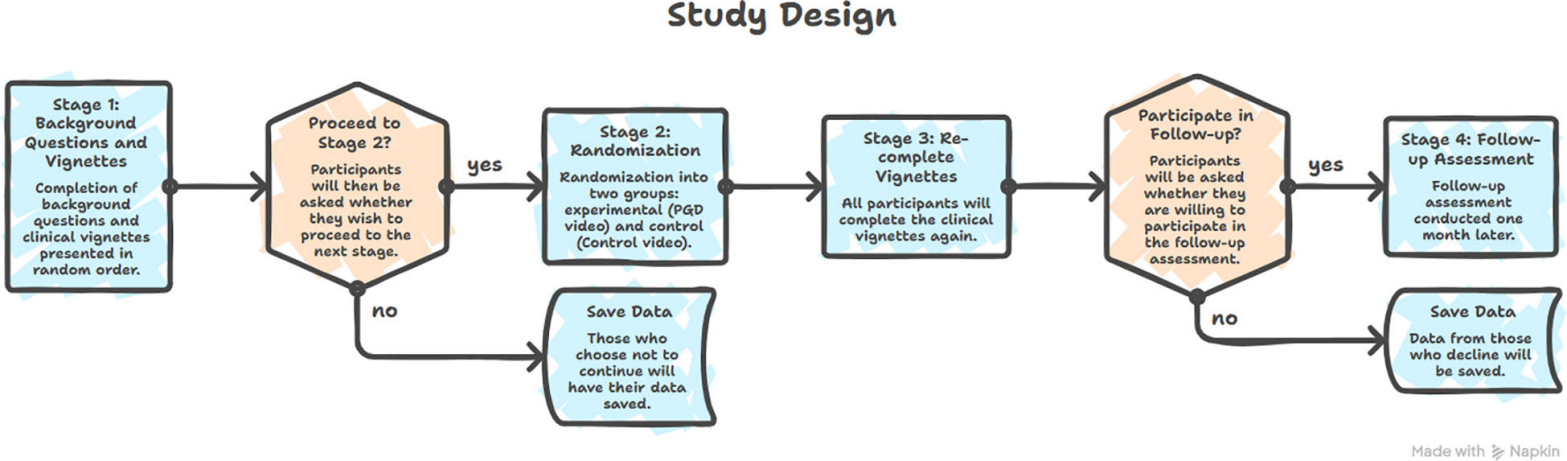
Study design.

In the first stage, participants will complete the online questionnaire, including background items and seven vignettes, estimated to take six minutes. The case vignettes will be presented in a randomized order for each participant to control potential order effects. Upon completing the questionnaire up to this point, participants will be asked to click the ‘Submit’ button, after which they will be prompted to indicate whether they wish to continue to the next phase of the study. This procedure is intended to ensure that the data of participants who choose not to continue are preserved and not lost. Participant responses across study stages will be linked using an identifier code, allowing for repeated-measures analyses while maintaining participant anonymity. In the second stage, participants will be individually randomized (1:1) via a computer-generated sequence in a partially participant-blinded, assessor-blinded controlled trial to either the experimental group (PGD video) or the control group (general bereavement video). In the third stage, all participants will re-answer the seven vignettes to assess the intervention’s effect on knowledge and application, taking approximately four minutes. Following completion of this stage, all participants will be asked whether they are willing to participate in a follow-up assessment. Those who consent will, at one month, receive an automated link to complete the clinical vignettes again to assess retention of the intervention effects (fourth stage). An overview of the study design is shown in **Figure 1**.

Upon completion of their participation, participants in the control group will be provided access to the PGD video to ensure equitable access to professional information; this will occur immediately after the third stage for those who do not consent to follow-up, and after completion of the one-month follow-up assessment for those who do. All collected data will be stored anonymously and encrypted, with access limited to authorized researchers for study purposes only. Participants will remain anonymous throughout, and results will be reported only in aggregate form.

*Data Analysis:* To test the study hypotheses, data analysis will include descriptive and inferential statistics, including simple linear regression, one-way analysis of covariance (ANCOVA) controlling for relevant background variables (e.g., levels of professional training), and mixed-design repeated measures ANCOVA.

*Feasibility assessment:* A preliminary feasibility assessment (N = 20 physicians, of whom 16 were senior physicians) was conducted to evaluate the study procedures and materials. Baseline performance on the clinical vignettes yielded a mean correct response rate of 61.4%, broadly consistent with prior reports in the literature (e.g (27). Overall, 12 participants completed both pre- and post-intervention assessments. Preliminary patterns suggested a potential intervention effect: in the control group, correct responses increased from 59.1% pre-intervention to 65.7% post-intervention, whereas in the experimental group, correct responses increased from 63.3% to 83.7%. These observations should be interpreted with caution given the small sample size and exploratory nature of this feasibility assessment and are presented solely to support the practicality and potential utility of the study design.

## Discussion

Although loss and bereavement are universal experiences that every clinician is likely to encounter in practice, these issues often receive only marginal attention in professional training, particularly among practitioners who work with older adults. One potential consequence of inadequate training is difficulty in identifying situations that fall outside the normative range of responses to loss, such as PGD.

Because PGD was only recently incorporated into diagnostic manuals, it is highly likely that many physicians are still insufficiently familiar with its diagnostic criteria. This lack of familiarity may result in under-recognition or misdiagnosis of the disorder and, consequently, in treatment that does not address its specific features. These challenges are especially evident among older adults, for whom the assessment and management of psychological distress are often suboptimal.

This is the first study in Israel to systematically examine the implementation of PGD diagnosis among physicians. In addition to providing a novel up-to-date overview, the study will also evaluate the effectiveness of a brief, targeted intervention in enhancing knowledge in this area. Key strengths of the present study lie in several features that extend prior work. The study focuses exclusively on physicians, a group that has been underrepresented in previous research (27–30); employs a pre–post design incorporating a brief intervention, in contrast to the more time-intensive approaches typically used in earlier studies (27, 30); and includes a follow-up assessment to examine the persistence of learning over time. It further focuses specifically on bereavement in older adults and is conducted in a context characterized by high exposure to loss and trauma (31), thereby enhancing its clinical relevance. Finally, it provides an updated assessment following the formal inclusion of PGD in the ICD-11 and approximately four years after the initiation of its global implementation.

Findings are expected to provide an empirical foundation for policy development, guide professional training, and contribute to the design of prevention programs and clinical guidelines to enhance PGD diagnosis and treatment. Moreover, participation in the study itself is also expected to increase physicians’ awareness of the diagnosis, thereby contributing to improved quality of care. In the long term, applying the study’s conclusions may improve clinical processes, reduce costs associated with underdiagnosis or inappropriate treatment, and provide data for future monitoring of implementation trends and continuous improvement. Additionally, the findings are expected to serve as a foundation for replication studies among other professional groups and to facilitate international comparisons, thereby advancing global knowledge and standardization in PGD diagnosis and care.

### Practical and operational considerations

In conducting this study, several practical and operational considerations should be noted. Recruiting physicians and residents across multiple specialties may present logistical challenges, particularly given their demanding schedules. In addition, convenience sampling may introduce potential selection bias. The use of an online questionnaire increases feasibility but may introduce variability in participants’ level of engagement and attention. Additionally, the repeated-measure design, which requires completion of clinical vignettes both before and after the intervention, relies on participants’ willingness to complete all components in a single session and may therefore affect response rates. Potential biases related to self-reported data should also be acknowledged, as they may influence the accuracy of participants’ responses. Despite these challenges, the study design is considered practical and feasible and is well suited for evaluating changes in diagnostic understanding following a brief educational intervention.

To address these challenges, several strategies have been incorporated into the study design. To enhance recruitment feasibility, formal collaboration agreements were established with key professional leaders in the relevant medical fields (including the Chair of the Israeli Geriatrics Association, the Chief Geriatrician of the largest health maintenance organization in Israel, and others), supporting coordinated dissemination of the study invitation and increasing the likelihood of broad participation. To mitigate potential selection bias, the study will aim to ensure balanced representation across medical specialties and levels of professional training. In order to reduce limitations associated with the online format and potential lapses in attention, the intervention will be delivered through a concise animated video, an evidence-based educational modality shown to improve engagement and cognitive processing, as previously described in the Methods section. The questionnaire was intentionally kept brief to minimize participant effort, and after completing the initial knowledge-mapping stage, participants will have the option to discontinue or proceed with the intervention, with responses from the first stage retained for all participants. Follow-up analyses will examine potential differences between those who continue and those who discontinue, allowing identification of any systematic attrition patterns. Finally, to reduce self-report bias, the study is conducted anonymously, which is expected to limit socially desirable responding. In addition, the use of clinical vignettes provides a performance-based assessment of diagnostic application, thereby reducing reliance on self-reported knowledge and strengthening the validity of the evaluation.

Ultimately, this study seeks to reduce suffering and enhance both quality of life and psychological resilience among older adults. At a time when scientific focus is often on advanced technologies and cutting-edge research, the value of this work lies in its simplicity and practical orientation. Its strength stems from translating current scientific knowledge into clear, actionable tools that can meaningfully improve clinical practice and the quality of care delivered to older patients.

## References

[1] World Health Organization . International classification of diseases for mortality and morbidity statistics (11th revision). Geneva: WHO (2021).

[2] American Psychiatric Association . Diagnostic and statistical manual of mental disorders. Arlington: American Psychiatric Association (2022).

[3] Reynolds CF Cozza SJ Maciejewski PK Prigerson HG Shear MK Simon N . Grief and prolonged grief disorder. Reynolds CF Cozza SJ Maciejewski PK Prigerson HG Shear MK Simon N , editors. Washington, DC: American Psychiatric Pub (2023).

[4] Cunningham J Shevlin M Cerda C McElroy E . ICD-11 prolonged grief disorder, physical health, and somatic problems: A systematic review. Clin Psychol Eur. (2025) 7:e14351. doi:10.32872/cpe.14351 40177338 PMC11960567

[5] Killikelly C Smith KV Zhou N Prigerson HG O'Connor MF Kokou-Kpolou CK . Prolonged grief disorder. Lancet. (2025) 405:1621–32. doi:10.1016/S0140-6736(25)00354-X 40254022

[6] Prigerson HG Maciejewski PK . Prolonged grief disorder: Detection, diagnosis, and approaches to intervention. World Psychiatry. (2024) 23:361. doi:10.1002/wps.21228 39279408 PMC11403192

[7] Shear MK Ghesquiere A Glickman K . Bereavement and complicated grief. Curr Psychiatry Rep. (2013) 15:406. doi:10.1007/s11920-013-0406-z 24068457 PMC3855369

[8] Buur C Zachariae R Komischke-Konnerup KB Marello MM Schierff LH O'Connor M . Risk factors for prolonged grief symptoms: A systematic review and meta-analysis. Clin Psychol Rev. (2024) 107:102375. doi:10.1016/j.cpr.2023.102375 38181586

[9] Domingue BW Duncan L Harrati A Belsky DW . Short-term mental health sequelae of bereavement predict long-term physical health decline in older adults: US Health and Retirement Study Analysis. J Gerontol B Psychol Sci Soc Sci. (2021) 76:1231–40. doi:10.1093/geronb/gbaa044 32246152 PMC8200357

[10] Ennis J Majid U . Death from a broken heart”: A systematic review of the relationship between spousal bereavement and physical and physiological health outcomes. Death Stud. (2021) 45:538–51. doi:10.1080/07481187.2019.1661884 31535594

[11] Prior A Fenger-Grøn M Davydow DS Olsen J Li J Guldin MB . Bereavement, multimorbidity and mortality: A population-based study using bereavement as an indicator of mental stress. Psychol Med. (2018) 48:1437–43. doi:10.1017/S0033291717002380 28851470

[12] Becker CB Taniyama Y Kondo-Arita M Sasaki N Yamada S Yamamoto K . Unexplored costs of bereavement grief in Japan: Patterns of increased use of medical, pharmaceutical, and financial services. OMEGA (Westport). (2021) 83:142–56. doi:10.1177/0030222821992193 33530889 PMC7983339

[13] Lichtenthal WG Roberts KE Donovan LA Breen LJ Aoun SM Connor SR . Investing in bereavement care as a public health priority. Lancet Public Health. (2024) 9:e270-e274. doi:10.1016/S2468-2667(24)00030-6 38492580 PMC11110717

[14] Van den Berg GJ Lundborg P Vikström J . The economics of grief. Econ J. (2017) 127:1794–832. doi:10.1111/ecoj.12399 40046247

[15] Callahan CM Bateman DR Wang S Boustani MA . State of science: Bridging the science-practice gap in aging, dementia and mental health. J Am Geriatr Soc. (2018) 66:S28–35. doi:10.1111/jgs.15320 29659003 PMC6690193

[16] Dashti S Najafi TF Mohammadzadeh F Kalat AR Bahri N . Knowledge level of health care providers about complicated grief during the COVID-19 pandemic. Iran J Psychiatry. (2022) 17:154–61. doi:10.18502/ijps.v17i2.8905 36262764 PMC9533357

[17] Devita M De Salvo R Ravelli A De Rui M Coin A Sergi G . Recognizing depression in the elderly: Practical guidance and challenges for clinical management. Neuropsychiatr Dis Treat. (2022), 2867–80. doi:10.2147/NDT.S347356 36514493 PMC9741828

[18] Kvalbein-Olsen LC Aakhus E Haavet OR Werner EL . Unrecognised depression among older people: a cross-sectional study from Norwegian general practice. BJGP Open. (2023) 7:1. doi:10.3399/BJGPO.2022.0135 36564082 PMC10354319

[19] Bar-Tur L . The challenge of aging: Mental health, assessment, and treatment (Hebrew). Jerusalem: Joint Israel-Eshel Publishing (2019).

[20] Hao F Qiu F Liang Z Li P . Psychotherapies for prolonged grief disorder in adults: A systematic review and network meta-analysis. Asian J Psychiatry. (2024) 99:104133. doi:10.1016/j.ajp.2024.104133 38970900

[21] Shear MK Reynolds CF Simon NM Zisook S Wang Y Mauro C . Optimizing treatment of complicated grief: A randomized clinical trial. JAMA Psychiatry. (2016) 73:685–94. doi:10.1001/jamapsychiatry.2016.0892 27276373 PMC5735848

[22] Kakarala SE Roberts KE Rogers M Coats T Falzarano F Gang J . The neurobiological reward system in prolonged grief disorder (PGD): A systematic review. Psychiatry Res Neuroimaging. (2020) 303:111135. doi:10.1016/j.pscychresns.2020.111135 32629197 PMC7442719

[23] Amsalem D Haim-Nachum S Lazarov A Levi-Belz Y Markowitz JC Bergman M . The effects of war-related experiences on mental health symptoms of individuals living in conflict zones: A longitudinal study. Sci Rep. (2025) 15:889. doi:10.1038/s41598-024-84410-3 39762464 PMC11704351

[24] Levi-Belz Y Groweiss Y Blank C Neria Y . PTSD, depression, and anxiety after the October 7, 2023, attack in Israel: A nationwide prospective study. EClinicalMedicine. (2024) 68:102418. doi:10.1016/j.eclinm.2023.102418 38586476 PMC10994954

[25] Buch SV Treschow FP Svendsen JB Worm BS . Video- or text-based e-learning when teaching clinical procedures? A randomized controlled trial. Adv Med Educ Pract. (2014) 5:257–62. doi:10.2147/AMEP.S62473 25152638 PMC4140394

[26] Koch M Günster SA Widder A Seyfried F Germer CT Backhaus J . Improved learning gain in medical students by using animated whiteboard-videos in comparison to textbooks in surgery. J Med Educ Curric Dev. (2024) 11:23821205241262684. doi:10.1177/23821205241262684 38882026 PMC11179544

[27] Kobak K Shear MK Skritskaya NA Bloom C Bottex G . A web-based therapist training tutorial on prolonged grief disorder therapy: Pre-post assessment study. JMIR Med Educ. (2023) 9:e44246. doi:10.2196/44246 36972105 PMC10131787

[28] Lichtenthal WG Maciejewski PK Demirjian CC Roberts KE First MB Kissane DW . Evidence of the clinical utility of a prolonged grief disorder diagnosis. World Psychiatry. (2018) 17:364. doi:10.1002/wps.20544 30229568 PMC6127759

[29] Dodd A Guerin S Delaney S Dodd P . Complicated grief knowledge, attitudes, skills, and training among mental health professionals: A qualitative exploration. Death Stud. (2022) 46:473–84. doi:10.1080/07481187.2020.1741048 32238122

[30] Jankauskaite G O’Brien KM Yoo SK Yang N Yoon S Bang J . Improving grief counseling: Enhancing the education and confidence of psychology graduate students. J Loss Trauma. (2025) 30:515–39. doi:10.1080/15325024.2024.2402393 37339054

[31] Malkinson R Manevich A Rubin SS Witztum E . Mass trauma, multiple losses, and the application of the Two-Track Model of Bereavement in the context of war: Assessment from a systemic-ecological perspective. Omega (Westport). (2025), 00302228251337127. doi:10.1177/00302228251337127 40279285

